# Drivers of the Distribution of Fisher Effort at Lake Alaotra, Madagascar

**DOI:** 10.1007/s10745-016-9805-1

**Published:** 2016-02-01

**Authors:** Andrea P. C. Wallace, Julia P. G. Jones, E. J. Milner-Gulland, Graham E. Wallace, Richard Young, Emily Nicholson

**Affiliations:** Department of Life Sciences, Silwood Park Campus, Imperial College London, Ascot, SL5 7PY UK; Frankfurt Zoological Society, PO Box 450189, Musakanya Drive, Mpika, Zambia; School of the Environment, Natural Resources and Geography, Bangor University, Bangor, LL57 2UW UK; Durrell Wildlife Conservation Trust, Les Augres Manor, Trinity, Jersey, JE3 5BP Channel Islands UK; Department of Biology & Biochemistry, University of Bath, Bath, BA2 7AY UK; Deakin University, Geelong, Australia. School of Life and Environmental Sciences, Centre for Integrative Ecology, (Burwood Campus), 221 Burwood Highway, Burwood, VIC 3125 Australia; Department of Zoology, University of Oxford, South Parks Road, Oxford, OX1 3PS UK

**Keywords:** IFD, Fisher spatial behaviour, Multi-habitat fishery, Adaptation, Risk, Madagascar

## Abstract

Understanding how fishers make decisions is important for improving management of fisheries. There is debate about the extent to which small-scale fishers follow an ideal free distribution (IFD) – distributing their fishing effort efficiently according to resource availability rather than being influenced by social factors or personal preference. Using detailed data from 1800 fisher catches and from semi-structured interviews with over 700 fishers at Lake Alaotra, the largest inland fishery in Madagascar, we show that fishers generally conform to IFD. However, there were differences in catch: effort relationships between fishers using different gear types as well as other revealing deviations from the predictions of IFD. Fishers report routine as the primary determinant of their choice of fishing location, explaining why they do not quickly respond to changes in catch at a site. Understanding the influences on fishers’ spatial behaviour will allow better estimates of costs of fishing policies on resource users, and help predict their likely responses. This can inform management strategies to minimise the negative impacts of interventions, increasing local support for and compliance with rules.

**Electronic supplementary material:**

The online version of this article (doi:10.1007/s10745-016-9805-1) contains supplementary material, which is available to authorized users.

## Introduction

Inland fisheries are widely recognised as significant sources of food and income for rural communities but most are fully exploited or overfished (FAO 2010; Welcomme 2011). Understanding fisher behaviour is critical to designing interventions that account for how fishers may respond to management in order to minimise the adverse impacts of interventions, and thereby increase the likelihood of fisher support and compliance (Wilen *et al*. 2002; Salas and Gaertner 2004; Cinner *et al*. 2008). To date, most fisheries literature focuses on commercial fishing fleets in developed countries whereas comparatively little is known about the complexities of subsistence or artisanal fisheries in developing countries (Welcomme *et al*. 2010). However, many of the factors that influence fisher decision-making and behaviour in commercial fisheries may be applicable in subsistence or artisanal contexts: economic factors such as risk strategy, access to gear or vessels, and fish prices (van Oostenbrugge *et al.*^2001^; Cinner and McClanahan 2006; Tidd *et al.*^2011^); biological factors such as fish densities and distribution (Gillis *et al*. 1993; Abernethy *et al*. 2007); environmental factors such as weather and human activity (Cinner and McClanahan 2006; Daw *et al.*^2011^); social and institutional factors such as traditions and spatial or temporal restrictions (Pálsson and Durrenberger 1990; Bjarnason and Thorlindsson 1993; Dinmore *et al*. 2003); as well as fisher-specific factors such as gear or bait preferences, effort, and fishing experience and ability (Parker and Sutherland 1986; Christensen and Raakjær 2006; Smith and Zhang 2007).

Rational choices and utility maximisation are primary tenets of traditional economic theory (Morse 1997; Güth 2008), and models based on these tenets have frequently been applied within fisheries research to explain fisher decision-making and behaviour (Holland 2008; Daw *et al.*^2011^). Such models assume that fishers have complete knowledge of fishery characteristics and use this information to make fishing decisions to maximise their personal utility, with profit often used as a proxy for utility (see Daw *et al.*^2011^). However, the applicability of microeconomic theory has been challenged by anthropologists (e.g., Ryan and Bernard 2006; Miller *et al*. 2014) and behavioural economists (e.g., Gelcich *et al*. 2007; Hastie and Dawes 2010), based on empirical evidence for how decisions are *actually* made rather than how they *should* be made. Accordingly, it is now increasingly recognised that fishers’ strategies or choices can vary considerably among individuals and involve a range of compromises that drive their patterns of fishing behaviour (Abernethy *et al*. 2007; Daw 2008; Holland 2008).

The concept of ideal free distribution (IFD) was first developed as a model of how animals distribute themselves among several patches of resources, the number of individuals being proportional to the amount of resources available at each location (Fretwell and Lucas 1969). The term ‘ideal’ assumes that harvesters have accurate knowledge of the distribution of targeted resources (such as fish species) and the term ‘free’ assumes that resource users (fishers) are able to move between locations without constraint (Kacelnik *et al.*^1992^; Gillis 2003). IFD has been applied to small-scale fishers but there is debate as to the extent to which it is a useful model to explain decision making of human harvesters (Abernethy *et al*. 2007), since other social or individual factors may be more important predictors of fishing behaviour.

For this study we examined fisher behaviour at Lake Alaotra, the largest lake in Madagascar and base for the nation’s most productive inland fishery (Andrianandrasana *et al*. 2005). We use information from in-depth semi-structured interviews and structured catch interviews with fishers from Anororo village to address four key questions:

1. What appear to be the main drivers of effort and choice of fishing location?
2. Do fishers follow an ideal free distribution in their effort?
3. What appear to be the drivers of deviations from IFD?
4. Under what circumstances do fishers change their fishing behaviour, particularly location?

We consider our results in the light of current and planned conservation interventions, which include no take zones and gear restrictions.

## Methods

### Study Site

**Fig. 1:**
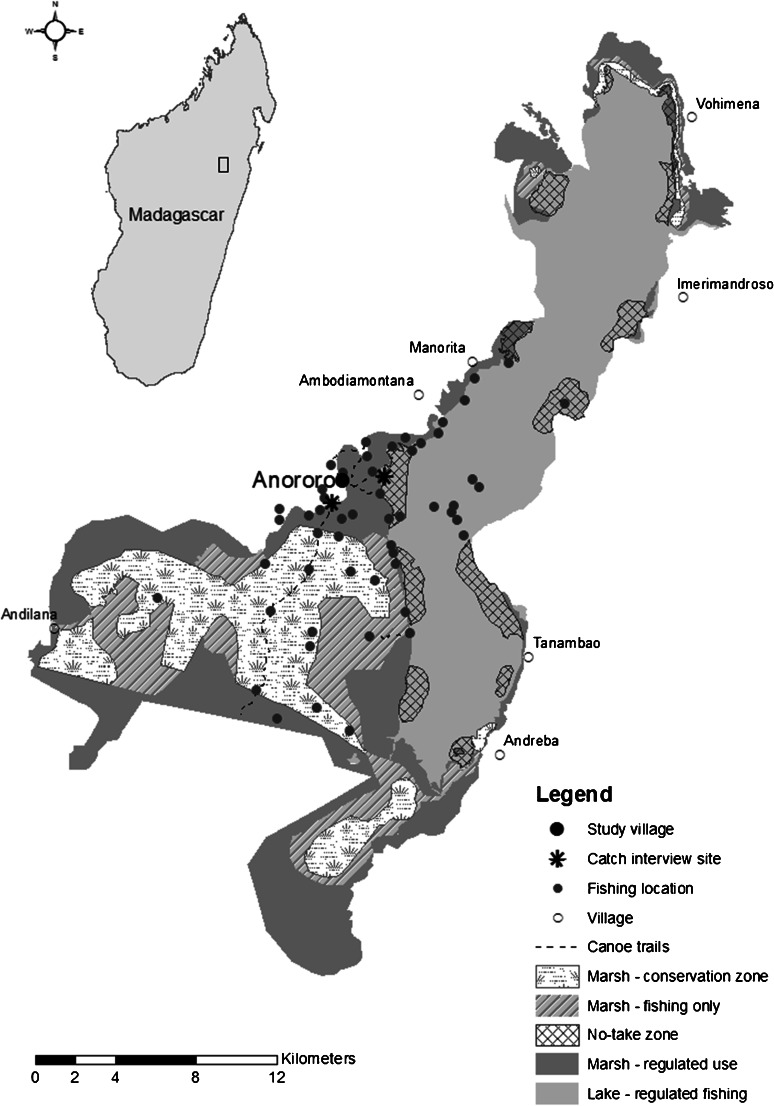
Map of Lake Alaotra showing management zones within the lake and adjacent marsh, catch interview sites, and the centroids of fishing locations used by local fishers as recorded in the catch interview data

The Alaotra wetland in northeast Madagascar is internationally recognised as an important area for biodiversity conservation. It was declared a Ramsar site in September 2003 (Ramanampamonjy *et al.*^2003^) and gazetted as a new protected area by the government of Madagascar in 2007 (Andrianandrasana 2009). Lake Alaotra covers 200 km^2^ and has a seasonal maximum depth of 4 m (Moreau 1979; Vanden Bossche and Bernacsek 1991; Ferry *et al*. 2009). The marsh adjoining the lake covers 230 km^2^ (Andrianandrasana *et al*. 2005; Ferry *et al*. 2009). Both the lake and the marsh areas are used by fishers (Fig. 1). There are two main climatic seasons: the wet season occurs from December to April and is hot with heavy rain and rising water levels, while the dry season from May to November is cooler and water levels decrease. There is up to two metres difference in water level between the high in March and the low in November (Moreau 1979; Ferry *et al*. 2009).

The human population in the Lake Alaotra area has increased from 109,000 in 1960 (Pidgeon 1996) to 550,000 in 2003 (Andrianandrasana *et al.*^2005^). The number of fishers operating in Lake Alaotra increased from approximately 1000 in the 1960s to over 4000 by 1989 (Wilmé 1994) and continues to increase with over 7000 fishers currently operating in the lake (H. Andrianandrasana, DWCT, pers. comm.), adding further pressure on already heavily exploited and overexploited fish stocks (Vanden Bossche and Bernacsek 1991; Pidgeon 1996).

Fisheries management authority for Lake Alaotra is vested in the Service Régional de la Pêche et des Ressources Halieutiques (Fisheries Service). Local fishers are represented by a Federation of Fishers that is intended to work with the Fisheries Service to co-manage resources by developing, implementing, and monitoring fishing regulations. In practice, the Federation of Fishers is perceived by many fishers as an extension of the Fisheries Service and therefore distrusted (APCW pers. obs.). There is a general lack of enforcement and poor compliance with all regulations, which were drawn up with limited consideration or knowledge of impacts on local fishers (Wallace *et al*. 2015). Consequently, the Lake Alaotra Management Plan is largely viewed as a ‘paper park’ that is failing to meet conservation goals (Fig. 1).

Anororo village is a relatively large community of approximately 8000 people on the western edge of Lake Alaotra within the Alaotra-Mangoro region of Madagascar (PCD 2004), adjacent to marsh and lake habitat, and was selected for (i) its large population of fishers using a variety of habitats and fishing methods, (ii) proximity to current and planned fishery management interventions, and (iii) local dependence on fishery resources for subsistence and commercial activity. Current conservation interventions are based on the 2006 Lake Alaotra Management Plan, which aimed to improve the sustainability of the fishery, reduce pressures on the wetland, and conserve rare bird and mammal species, particularly the Critically Endangered Alaotran gentle lemur (Razanadrakoto and Rafaliarison 2005).

The subsistence and cash economy of Anororo centres on fishing and rice cultivation. Although some households engage in both activities, we targeted people who are primarily fishers with very few, if any, alternative sources of food and income. Anonoro-based fishers are male and almost exclusively (99 %) from the Sihanaka ethnic group. Those we interviewed were broadly representative of the landless population in Anororo, who have lower incomes and fewer assets than local land-owning rice farmers, in terms of ethnicity, age distribution, level of education, and number of household members supported (Le Courtois 2010).

Fishers operate independently from dugout canoes and use a broad range of gear types including traps, gill nets, cast nets, and line & hook. Traps and line & hook are used passively overnight with usually 24 h between fish collections. Gill nets were observed being used in three ways: (i) passively overnight, (ii) passively while waiting, and (iii) actively. Cast nets are used actively or passively with bait. Fishing occurs in lake, marsh, and lake-marsh edge habitats. The main market for fish is commercial buyers from Antananarivo, Madagascar’s capital; local market and bicycle collectors also operate in the village.

### Data Collection

Data were collected in June and July 2009 and from October 2009 to December 2010, comprising two dry seasons (2009 and 2010) and one wet season (2010). We conducted three types of interviews with Anororo-based fishers: structured catch interviews (*n* = 1800); semi-structured background interviews (*n* = 405); and subsequent follow-up interviews (*n* = 221). Local research assistants conducted interviews in Malagasy and translated responses into French.^1^ All interviews were conducted opportunistically with fishers who were willing to participate. Fishers were informed that participation was voluntary and that their identities and responses would not be shared with anyone.

Catch interviews were conducted at two sites (one on the lake and one in the marsh on the main thoroughfares to the village where buyers wait) prior to fishers selling their catch. Fishers do not return to the village with their catch if they intend to sell it, which was the case for most fishers. Background interviews were conducted by walking through all areas of the village and approaching people who had some involvement in fishing (identified with help from local guides and key informants). Follow-up interviews were conducted 11 to 12 months later, and only with fishers who had participated in background interviews. No fishers refused to be re-interviewed. A total of 784 fishers, approximately 85 % of fishers in the village, participated in one or more types of interview. A total of 110 fishers participated in all three types of interviews. Respondent codes were assigned to all participating fishers to preserve their anonymity (Bernard 2002) as well as to triangulate the data.

#### Catch Interviews

(5 to 10 min) comprised a series of questions about fishing activity that day, including the fishing location, gear type, and effort used. A mapping exercise conducted with senior and experienced fishers, followed up with visits to take GPS waypoints, provided locations of locally important fishing areas (Fig. 1; Wallace 2012). A total of 537 individual fishers participated in catch interviews; 248 fishers (46 %) were interviewed more than once. Catch interviews also included counting and measuring the fish caught (*n* = 27,064). Total catch weights were estimated using species-specific length-weight relationships (Wallace 2012).

#### Background Interviews

collected information on demographics, reliance on fishing for livelihood, and fishing behaviour including type of gear and fishing location(s) used. A total of 158 fishers participated in catch interviews as well as background interviews.

#### Subsequent Follow-Up Interviews

collected information on perceptions of the status of the fishery and management interventions, as well as how fishers’ spatial distribution has changed over time, particularly whether they added or dropped locations since the previous background interview and the reasons for changes.

### Data Analysis

We first analysed background interviews to identify key demographic factors that best explain variation in effort across fishers. Second, we plotted data on catch and effort for fishing locations for comparison with the 1:1 prediction of the catch:effort relationship expected under IFD, and examined in detail those locations that deviated from IFD. Finally, we used background and follow-up interview data to examine drivers of distribution and changes in distribution of effort. Quantitative data, including frequencies and proportions, were analysed using R version 2.14.2 (R Development Core Team 2012).

#### Analysis of Fisher Effort

To identify different groups of fishers and determine the factors influencing fisher effort, measured as time spent fishing, we analysed data from background interviews with fishers (*n* = 405) using a negative binomial generalised linear model (GLM). Five socioeconomic characteristics of fishers were assessed as explanatory variables: (1) age category, (2) total number of people supported in the household, (3) level of education (up to primary school or secondary school and above), (4) whether the fisher’s household has an alternative livelihood or source of income, and (5) type of gear (e.g., traps, gill nets) used for fishing. Time spent fishing is a meaningful measure of fisher effort in both biological and economic terms, and allows for comparison between gear types as well as between fishing locations at varying distances from the village (Abernethy *et al*. 2007; Daw *et al.*^2011^). Our measure of effort and analysis of IFD did not include travel time, due to potentially confounding factors such as other activities undertaken en-route to fishing location as well as individual differences in paddling speed related to age and/or physical fitness. Previous research within the Lake Alaotra system confirms time spent fishing influences catch weight but travel time does not (Wallace *et al*. 2015).

Years of fishing experience was significantly and strongly positively correlated with fisher age (*r* = 0.81, *p* < 0.0001) and therefore not included as an explanatory variable. Almost all fishers interviewed (99 %) were from the Sihanaka ethnic group, and 98 % had lived in Anororo since birth. Most interviewees (86 %) stated that fishing was their primary livelihood, and many also had an alternative source of income during the calendar year. Lack of variation in these factors meant it was not possible to examine their influence on fisher effort and they were therefore not included in the analysis.

We used Akaike’s Information Criterion (AIC) model selection to rank models and quantify the magnitude of difference between them, and model averaging to determine model-averaged coefficients (Burnham and Anderson 2002; Bolker *et al*. 2009). The global model was run using the MASS package in R; the MuMIn package was used for model comparison and averaging. Following Burnham and Anderson’s (2002) rule of thumb, all models where AIC differences were less than four were included in the candidate set of models for model averaging. AIC differences of <4 was chosen because the weight or support for subsequent models decreased considerably at this point. No single model was clearly superior to others in the candidate set of models, suggesting that model averaging would provide a more robust understanding of the system and reduce model selection bias effects (Burnham and Anderson 2002).

#### Ideal Free Distribution

IFD states that harvesters will distribute themselves in relation to resource availability and predicts that the proportion of aggregate effort will be equal to the proportion of aggregate catch at each location (Fretwell and Lucas 1969). We analysed data from catch interviews for a total of 1757 fishing trips by 515 individual fishers; separate analyses were conducted for a sub-sample of 788 fishing trips by 151 individual fishers who had also participated in background interviews. Catch interviews where no fish were measured (*n* = 39) because fishers had sold their catch prior to interview, did not want to have their fish measured, or did not have time for their fish to be measured, were excluded from analyses. Interviews with fishers who had fished in the immediate vicinity of the village were infrequent (*n* = 4) and also excluded from analyses. Proportions of catch (measured as total weight caught) and effort (measured as total number of hours spent fishing) observed at fishing locations over the study period were calculated across all gear types and for each gear type. Deviation was calculated as proportion of catch divided by proportion of effort over the study period by gear type. A positive deviation (>1) occurs where proportion of catch exceeds proportion of effort; a negative deviation (<1) occurs where proportion of effort exceeds proportion of catch. We examined the attributes of particular sites that deviated from IFD to determine drivers of fisher behaviour.

#### Stated Drivers of Fisher Behaviour

Fisher responses to semi-structured interview questions were categorised into common themes for analysis. Response sample size varies according to whether background or follow-up interviews were conducted and because fishers sometimes gave vague or ambivalent responses that could not be categorised. Data are presented as the percentage of interviewees providing a particular response to questions regarding (a) reasons for choosing a fishing location, and (b) whether they changed location(s) chosen for fishing during the study period and why. We summarized differences among fisher groups and compared them using chi-square and Fisher’s exact tests. In particular we were interested in drivers of change in location, and whether fishers who changed did so because conditions had deteriorated (circumstances ‘pushed’ them out) or were perceived to have improved in the new location (‘pulled’ them to the new location). We hypothesized that if fishers truly conformed to IFD they would predominantly be ‘pulled’ into new locations for better catches in order to maximise returns per unit of effort.

## Results

### Drivers of Fishing Effort

Results from the negative binomial GLM indicated that gear type and number of people supported were significant predictors of time spent fishing (Table 1). Fisher age category, level of education, and presence of an alternative livelihood in the household were not significant explanatory variables. These results confirmed that gear type could be used to categorise fishers at a broad scale.

**Table 1:** Results of the negative binomial generalised linear model of fisher profile variables explaining fisher effort measured as time spent fishing

Explanatory variables	Estimate	SE	*z*	*P*
Intercept	5.9886	0.2931	20.397	<0.0001
Age category^a^				
Age25-34	−0.1793	0.1055	1.697	0.0897
Age35-44	0.0071	0.1074	0.066	0.9471
Age45-54	−0.1756	0.1186	1.478	0.1393
Age55+	0.0203	0.1261	0.161	0.8724
Total dependents	0.0406	0.0136	2.975	**0.0029**
Education^b^				
Secondary	0.0832	0.0449	1.852	0.0640
Alternative livelihood^c^				
Yes	0.0342	0.0591	0.578	0.5634
Gear type^d^				
Gill nets	0.7292	0.0481	15.148	**<0.0001**
Cast nets	1.1004	0.1332	8.250	**<0.0001**
Line & hook	1.4453	0.2766	5.218	**<0.0001**
Hand methods	0.3528	0.1581	2.228	**0.0259**

Baseline levels are ^a^‘Age15-24’, ^b^‘primary school education,’ ^c^‘no alternative livelihood,’ and ^d^‘traps.’ Significant values are in bold

The characteristics and fishing activity of fishers who had participated in catch interviews as well as background interviews (*n* = 151) were therefore grouped by gear type for comparison. The characteristics of fishing activity, in particular mean catch per trip in kilograms and mean effort (time spent fishing in hours) per trip, differed significantly across gear types (Table 2).

**Table 2:** Characteristics of fishers and their fishing activity by gear type

Characteristic	Gear type					ANOVA
	Trap	Gill net	Cast net	Line & hook	Hand methods	
Number of fishers in cluster (*n* = 151)^a^	88	58	5	4	9	–
Mean catch per trip (kg)	1.66	1.73	5.28	3.34	0.81	*F* = 5.74
	(±0.14)	(±0.27)	(±0.82)	(±1.19)	(±0.41)	***p*** **< 0.001**
Mean proportion of catch sold per trip	65 %	68 %	86 %	83 %	82 %	*F* = 2.04
	(±1.7)	(±3.2)	(±6.4)	(±6.7)	(±10.3)	p = 0.087
Mean effort (time spent fishing in hours) per trip	1.63	2.83	5.32	6.85	1.55	*F* = 89.22
	(±0.04)	(±0.19)	(±0.40)	(±1.44)	(±0.41)	***P*** **< 0.0001**
Mean one way distance travelled (km)	3.69	4.83	3.77	4.83	3.18	*F* = 9.30
	(±0.09)	(±0.18)	(±0.23)	(±1.49)	(±0.68)	***P*** **< 0.0001**
Mean years of fishing experience	18.9	16.8	31.3	17.5	13.4	*F* = 7.03
	(±0.52)	(±0.67)	(±2.44)	(±7.64)	(±2.57)	***P*** **< 0.0001**
Mean number of people supported in household	4.8	4.6	5.3	3.5	3.0	*F* = 4.82
	(±0.07)	(±0.10)	(±0.42)	(±1.04)	(±0.53)	***p*** **< 0.001**
Proportion with alternative livelihood	80 %	71 %	100 %	100 %	56 %	–

Standard errors (SE) are shown in parentheses. ANOVA results refer to differences between gear types for each characteristic (*df* = 4)

^a^Sums to >151 because 13 fishers used two gear types during the study

### Spatial Distribution of Fisher Effort

**Fig. 2:**
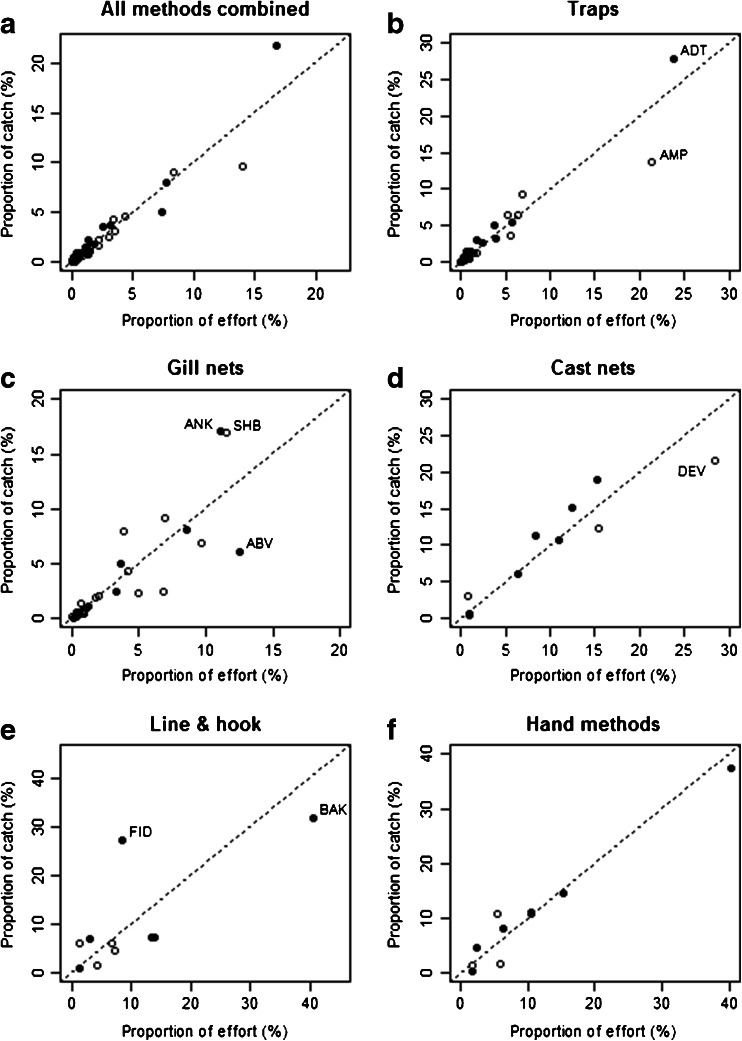
Proportions of catch and effort observed at fishing locations in Lake Alaotra over the study period (*n* = 1757 catch interviews with 515 individual fishers), calculated across all gear types and for each gear type. Catch was measured as total weight caught and effort was measured as total number of hours spent fishing at the location over the period. *Solid circles* represent fishing locations within restricted areas; *open circles* represent locations within non-restricted areas (see Table 3 for characteristics of each labelled location). The *dotted line* represents the 1:1 prediction of IFD

Across all fishing trips, irrespective of gear type and fisher identity, fishers appear to conform to IFD; the proportion of effort (i.e., time spent fishing) allocated to fishing locations is directly proportional to the proportion of catch derived from those locations (Fig. 2a). There are however differences in deviation from IFD amongst fishers using different gear types (Fig. 2b to f). Notably, trap fishers appear to adhere more to IFD than gill net fishers.

Heteroscedasticity within the dataset is clear, and considerably more variation occurs at fishing locations where proportions of catch and effort are high (Fligner-Killeen test, *χ*^2^ = 24.64, *df* = 6, *p* < 0.001). There were no explicit patterns or differences between years (2009 vs. 2010) to explain this deviation from IFD. Linear models to explore factors influencing deviation for each gear type were inconclusive. However, for each gear type, some general patterns can be drawn from the characteristics of locations that deviate particularly strongly from IFD (Table 3).

**Table 3:** Characteristics of locations with greatest deviation from the ideal free distribution by gear type

Location	Location characteristics				
	Index of deviation from IFD^a^	Distance from village (km)	Habitat	Restricted area	Number of fishers in sample
Traps					*292*
Andratsilanina (ADT)	1.17	4.1	Edge	Yes	58
Amparihy (AMP)	0.64	1.3	Marsh	No	66
Gill nets					*155*
Ankororo (ANK)	1.53	3.4	Edge	Yes	24
Sahabe (SHB)	1.47	6.9	Lake	No	30
Ambavasaha (ABV)	0.49	2.0	Edge	Yes	26
Cast nets					*25*
Deversoir (DEV)	0.76	4.1	Edge	No	10
Line & hook					*14*
Farihi ’i Daganera (FID)	3.19	5.5	Marsh	Yes	4
Lasin ’i Bakoto (BAK)	0.79	4.8	Edge	Yes	2

^a^Deviation is proportion of catch divided by proportion of effort over the study period by gear type. A positive deviation (>1) occurs where proportion of catch exceeds proportion of effort; a negative deviation (<1) occurs where proportion of effort exceeds proportion of catch

An important factor for location selection is convenience, i.e., distance travelled. For trap fishers, for example, although Andratsilanina (ADT; positive deviation) is distant from Anororo, it is very suitable for setting traps at the lake-marsh edge and perceived to have high fish abundance. In comparison, Amparihy (AMP; negative deviation) is characterised by extensive human traffic and degraded habitat due to close proximity to Anororo. Despite the relatively high level of habitat degradation, fishing, and other activities at Amparihy, fishers continued to fish in this location due to its proximity to the village, indicating that they value convenience more highly than potentially higher catches elsewhere. For gill net fishers Ankororo (ANK) and Sahabe (SHB), both deviating positively from IFD, are low-traffic areas lacking vegetation in which nets can be damaged, entangled, or dislodged, in contrast to Ambavasaha (ABV; negative deviation) which is closer to Anororo. Disproportionately high fishing activity, given the returns, at Ambavasaha indicates that gill-net fishers are risk averse when it comes to personal safety, often stating that fishing near the lake edge was safer than further offshore despite smaller catches. For all gear types, returns were generally better at sites more suitable for the gear and/or within a protected area. Persistence at sites with lower returns was mostly attributable to convenient proximity to Anororo.

Proportions of catch and effort were also compared to IFD for the sub-sample of fishers (*n* = 151) who also participated in background interviews (Fig. S1). The sub-sample conformed to IFD in a similar pattern to that for the larger sample of all fishers participating in catch interviews (*n* = 515). The total sample of fishers participating in background interviews (*n* = 405) was therefore used to further explore the drivers of fisher behaviour.

### Factors Influencing Fishers’ Spatial Behaviour

Although fishers participating in catch interviews used a single location per fishing trip, fishing locations were used adaptively according to changing conditions over the year; fishers use a median of two (range 1 to 6) locations over the calendar year (Wallace 2012). Almost all fishers participating in background interviews (98 % of 405) stated they had continued to fish at the same location(s) over the last 5 years. Routine was the most frequently cited reason to use a location, followed by perception of good catches at these sites (Table 4). Being familiar with or having good knowledge of a fishing location was also stated as important; however, only seven of these fishers cited a long family history of fishing at a location as a reason for using it. Typical responses by fishers when asked why they use their location(s) include: “*It is my habit and I don’t know other locations;*” “*I am used to getting mostly good catches at this location;*” “*My location puts me at ease for fishing;*” and “*I know the route from habit and do not have to fear getting lost.*”

**Table 4:** Reasons provided by fishers (*n*=403) for choosing fishing locations. The proportion of fishers stating each reason is grouped by gear type

Reason	Traps	Gill nets	Cast nets	Line & hook	Hand methods	All gear types
	(*n*=213)	(*n*=167)	(*n*=10)	(*n*=7)	(*n*=6)	(*n*=403)
	%	%	%	%	%	%^a^
Routine – Usual location. Always uses this or these locations.	73.7	80.2	100.0	71.4	33.3	76.7
Catch – Many fish are present, good catches, good quality fish and/or presence of specific target species.	20.2	22.8	10.0	28.6	16.7	21.1
Familiarity – Fisher has good knowledge of the location (e.g., how to get there, move around the location, and catch fish) and the location is appropriate for the fisher’s skills and ability. May have a long history of fishing there.	18.3	8.4	0.0	14.3	0.0	13.4
Suitability – Location has characteristics (e.g., water level or habitat) that suit the fisher’s gear type or manner of fishing (e.g., camps out). There are favourable environmental characteristics for fishing; calm (no wind), sheltered or protected, location can be used all year. Fisher preference.	10.3	9.6	40.0	0.0	50.0	11.4
Travel – Close to village or rice field. Allows time for other activities. Close to collectors who buy fish. Location is not clogged with invasive plants. Ease of travel, accessible.	10.3	4.8	0.0	14.3	50.0	8.4
Fishers – No or few thieves. No large seine nets that destroy gear. Camaraderie, enjoyable because friends fish there.	4.7	1.8	0.0	0.0	0.0	3.5

^a^Proportions sum to >100 % because 129 respondents (32 %) nominated multiple reasons

The vast majority (94 %) of fishers interviewed use traps or gill nets; therefore we focussed on responses from these two groups, for whom the reasons for selecting fishing locations differed significantly (Fisher’s exact test, *p* = 0.024). Specifically, a greater proportion of trap fishers cited familiarity and travel time as reasons for selecting a location (chi-square tests: familiarity *χ*^2^ = 11.79, *df* = 1, *p* < 0.001; travel time *χ*^2^ = 6.53, *df* = 1, *p* = 0.011). Reasons for selecting a fishing location did not differ between age categories (Fisher’s exact test, *p* = 0.597).

Despite the high degree of consistency in relation to location choice during background interviews (Table 4; routine), 81 % of fishers subsequently participating in follow-up interviews (*n* = 221) stated they were using a different set of locations since the background interview due to unusual seasonal changes. Fishers stated that low rainfall and extended cooler temperatures early in 2010 limited fish movement and growth, resulting in reduced stock and ultimately reduced catch sizes. Consequently, the most frequently cited reasons for fishers being pushed out of or pulled into different fishing locations were related to catch size, travel, and water level (Table 5). In most cases, factors that pushed fishers out of their preferred fishing location(s) were more powerful, indicating their reluctance to move otherwise, and is consistent with initial responses specifying routine as the main driver of location choice. Only travel-related factors pulled rather than pushed a higher proportion of fishers to a new location, which was always closer to Anororo. Reasons for switching locations did not differ among gear types or among age categories (Fisher’s exact tests, *p* = 0.742 and *p* = 0.420, respectively).

**Table 5:** Reasons provided by fishers (*n* = 178) for being pushed out of or pulled into other fishing locations. The number and proportion of fishers stating each reason are grouped for pushed and pulled

Reason	Pushed		Pulled		Total	
	n	%	n	%	n	%^a^
Catch – Catch and fish size. Pushed out of location due to poor catches or small fish size. Pulled into other locations for better catches or larger fish. Follow seasonal movement of fish; fishers follow fish movement to continue to have a catch.	49	27.5	17	9.6	66	37.1
Travel – Pushed out of previous locations because access became difficult due to invasive plants. Pulled in because of proximity to village, residence, or rice field, less travel time, or allowing time for other activities. May change seasonally or with second season rice cultivation activities in the marsh, due to age or health of fisher, with changes in personal circumstances, and/or may involve risk aversion.	12	6.7	24	13.5	36	20.2
Water level – Pushed out of locations due to unusually low seasonal water levels in 2010.	31	17.4	0	0	31	17.4
Fishers – Pushed out because of overcrowding or presence of thieves, or due to presence of methods that make it difficult to use their preferred gear. Pulled in because location is not crowded or less crowded, has fewer thieves, or recommended by other fishers.	16	9.0	14	7.9	30	16.9
Suitability – Pushed out because location characteristics change over time and become unsuitable for preferred fishing strategy. Pulled in because location characteristics are better suited to the fisher’s choice of gear or manner of fishing (e.g., camps out).	12	6.7	9	5.1	21	11.8
Other – Pushed out due to habitat degradation and/or poor water quality (e.g., invasive plants degrading fishing locations in the marsh, dirty or stinking water). Pulled in to trial or explore additional fishing location(s).	6	3.4	2	1.1	8	4.5

^a^Proportions sum to >100 % because 14 respondents (8 %) nominated multiple reasons

A fisher’s choice of location is influenced, and often constrained, by type of gear used (e.g., traps cannot be used in open lake habitat because they need to be fixed to supporting materials such as reeds to hold them in place). Only 4 % of fishers participating in background interviews (*n* = 405) had changed their gear type in the previous five years. Most fishers (80 %) specified routine and/or competence as the primary reasons for continuing to use their preferred type of gear. Representative statements include: “*It is my habit, I have used nets for a long time;*” “*I am competent with traps and do not know how to use other methods;*” “*I know how to make nets;*” and “*Traps are easy to use and are my tradition.*”

## Discussion

Examining drivers of fisher effort and behaviour, this study shows that Anororo-based fishers within Lake Alaotra’s small-scale fishery generally conform to IFD, and they choose locations throughout the lake and marsh they believe will provide good or consistent (but not necessarily maximal) catches. Departures from IFD are primarily tied to convenience and/or the distinctive environmental or anthropogenic characteristics of a fishing site, which often assume importance for gear-specific reasons. Our finding that trap fishers adhere more to IFD than gill net fishers is counter-intuitive – trap fishers can move location less easily because their traps are left overnight and not readily moved large distances, whereas gill net fishers can in theory choose their location on a daily basis.

Type of gear used is the major determinant of fishing effort and choice of fishing location. Gear-based variation in fisher effort and spatial distribution often characterises subsistence or artisanal fisheries, particularly in developing tropical countries (Abernethy *et al*. 2007; Daw *et al*. 2011; Hamerlynck *et al*. 2011). Environmental and habitat factors may constrain the type(s) of gear a fisher can use, or render some methods more suitable than others (see Welcomme 2001). Whereas variation in gear use often occurs to target different species (Gillis *et al.*^1993^; Abernethy *et al.*^2007^), for Anororo-based fishers this variation is mediated by differences in habitat within their fishing arena. For example, gill nets require relatively large open areas clear of obstructions, which are typically further from the village and involve greater travel time, while fishers using hand methods require shallow water and/or marsh habitat, which occur closer to the village. These gear-specific influences of environmental factors affect fishers’ choices of fishing location (and therefore spatial distribution), demonstrating that gear type is central to understanding fisher behaviour within multi-habitat fisheries.

Our finding that Anororo-based fishers generally conform to IFD contrasts with Abernethy *et al*. (2007) for artisanal Anguillan reef fishers, where the relatively substantial departures from IFD were linked to fisher age, experience, and target species, as well as type of gear. In our study, species were not explicitly targeted, fisher age or experience were not drivers of catch (see Wallace *et al*. 2015), and catch influenced fishers when selecting or changing fishing location(s). This suggests that fishers pursue rational strategies to the extent their knowledge permitted when distributing effort spatially, which is usually more characteristic of larger-scale commercial fisheries than artisanal systems (Branch *et al.*^2006^; Powers and Abeare 2009). Despite this, and in contrast to the central assumption of IFD that all individuals aim to optimise profits (Gillis 2003; Abernethy *et al*. 2007), fishers do not fish in ways to maximise returns. Rather, in line with equivalent findings by Béné and Tewfik (2001), Cabrera and Defeo (2001), Salas and Gaertner (2004), and Daw (2008), fishers’ decisions on effort distribution are mediated by multiple trade-offs including convenience, routine, gear usability and maintenance, or predictability of catch. Commonly preferred strategies are (a) spend more time fishing closer to home rather than invest that time travelling to distant locations or (b) continue fishing in a familiar location where catches are more predictable but sometimes small (see Swain and Wade (2003) for similar strategies for fishers of snow crab in the Gulf of St. Lawrence, Daw (2008) for lobster fishers in Nicaragua, and Teh *et al*. (2012) for small-scale fishers in Sabah, Malaysia, indicating that a broad range of fishers compromise or satisfice instead of optimising utility (Simon 1955; Foxon 2006)).

We took a rational choice approach to understanding fishers’ decisions, by assessing the extent to which fishers’ decisions about spatial distribution conform to IFD. However, the range of influences and trade-offs reported by fishers suggest further research using alternative approaches to understanding decision-making may provide additional insights (Gintis 2007). Similarly, it would be informative to examine fishers’ behaviour over time and under changing conditions (including the implementation of new management) to determine the degree to which drivers and patterns of behaviour are fixed or adaptive and the degree to which they can be generalised to other fisheries.

## Conclusion

The predominance of routine as a driving factor for Anororo-based fishers extends from choice of fishing location through to selection of gear and persistence with that type of gear. Specifically, fishers’ decisions about location and gear intertwine; once choices are made, persistence is bound by (i) familiarity with relevant site characteristics, (ii) competence with type of gear used, and (iii) perceived costs of changing location or gear. Provided catches remain adequate for the amount of effort invested, which depends on trade-offs with fishers’ interests other than catch, there is considerable inertia within the fishery and reluctance to change. Routine, habit, and/or familiarity with location or gear are increasingly identified as key factors in fisher decisions, such as in New England trawl fisheries (Holland and Sutinen 2000), urchin divers’ location choices in California (Smith 2005), preferred resource spaces by small-scale fishers in Malaysia (Teh et al. 2012), and gear use by Swedish fishers (Eggert and Tveteras 2004).

Catch size and travel costs (which interlink with water level and access to fishing locations) are most likely to motivate fishers to change their spatial behaviour. Poor catches and/or high travel costs frequently push changes in fishers’ spatial behaviour. The prospect of better catches and/or lower costs per se has less effect because of the constraining influences of routine and familiarity. These patterns show that although Anororo-based fishers conform generally to IFD and make rational spatial decisions, they are risk averse and sub-maximal catches suffice under conditions of uncertainty. A key implication of this is that these and similar fishers could be less responsive to purely economic incentives to modify their behaviour than commonly expected (see Holland 2008). By demonstrating that fishers’ behaviour is typically mediated by tradeoffs and fishers do not always maximise returns, our study makes an important contribution to our understanding of fisheries dynamics by evaluating and explaining fisher spatial behaviour at a scale relevant to conservation planning. This understanding could be used to advantage in fisheries management to ensure plans and actions accommodate resource-users’ behaviour; it could also inform the development of meaningful and realistic incentives to support interventions and comply with regulations.

From a fisheries management perspective it is pragmatic to understand and account for fisher behaviour collectively (Béné and Tewfik 2001; Holland 2008; Cinner *et al*. 2010). Our study suggests that trade-offs and variation in spatial behaviour within multi-gear artisanal fisheries may be best understood by grouping fishers according to their type of gear, and that IFD is a useful null hypothesis against which to examine differences between fishers in their distribution among locations. Our findings are likely to also be applicable for some commercial fisheries, particularly where artisanal and commercial fisheries overlap. Misunderstandings about diversity of interests and motivations influencing fishers’ spatial behaviour have often led to inappropriate and ineffective management interventions, or compromised compliance with regulations, by imposing greater costs on fishers than they are able to bear (Peterson and Stead 2011). Data from this study may be used with spatial planning tools and scenario analysis to inform development of reserve designs that account effectively for fisher behaviour, costs to fishers, and biodiversity goals.

Management actions are likely to have substantial impact in subsistence, artisanal, and developing-country settings because fishers will typically be socioeconomically highly invested in and dependent on fishing. These fishers will also probably be relatively poor (and hence vulnerable and less resilient to shocks), lack buffers to offset seasonal variation in catches and income, and have limited livelihood options (Hill 2011; Teh *et al*. 2012). Greater research attention should be afforded to understanding the relationships among fishers’ motivations, perceptions of the costs of management actions, and responses to those actions in order to recommend management actions and conservation interventions with minimal negative impacts for fishers who depend on the fishery for livelihood. A better understanding of these relationships could in turn increase fisher compliance and hence the effectiveness of such actions and interventions.

## Electronic supplementary material

Fig. S1 Proportions of catch and effort observed across all gear types at fishing locations in Lake Alaotra, calculated for all fishers who also participated in background interviews (n = 788 catch interviews with 151 individual fishers). The pattern shown is similar to Fig. 2a and so justifying the use of the background interview dataset for all future analysis. Catch was measured as total weight caught and effort was measured as total number of hours spent fishing at the location. Solid circles represent fishing locations within restricted areas; open circles represent locations within non-restricted areas. The dotted line represents the 1:1 prediction of IFD. (JPEG 17 kb)

High Resolution Image (TIF 673 kb)

## Footnotes

1 APCW is fluent in French and over the course of the study, acquired working knowledge of Malagasy. JPGJ is fluent in conversational Malagasy.
