# Supplementary figures and images for: Drivers of the Distribution of Fisher Effort at Lake Alaotra, Madagascar
Source: Hum Ecol Interdiscip J. 2016 Feb 1;44:105–17. doi: 10.1007/s10745-016-9805-1 (PMC4757614; doi:10.1007/s10745-016-9805-1)

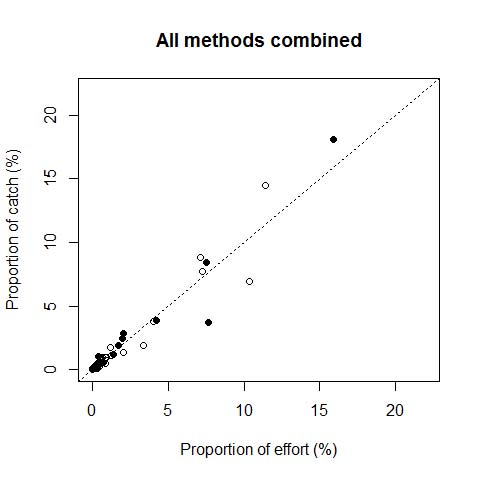

Supplement: Supplementary file 1 — Proportions of catch and effort observed across all gear types at fishing locations in Lake Alaotra, calculated for all fishers who also participated in background interviews (n = 788 catch interviews with 151 individual fishers). The pattern shown is similar to Fig. 2a and so justifying the use of the background interview dataset for all future analysis. Catch was measured as total weight caught and effort was measured as total number of hours spent fishing at the location. Solid circles represent fishing locations within restricted areas; open circles represent locations within non-restricted areas. The dotted line represents the 1:1 prediction of IFD. (JPEG 17 kb) [file 10745_2016_9805_Fig3_ESM.jpg]

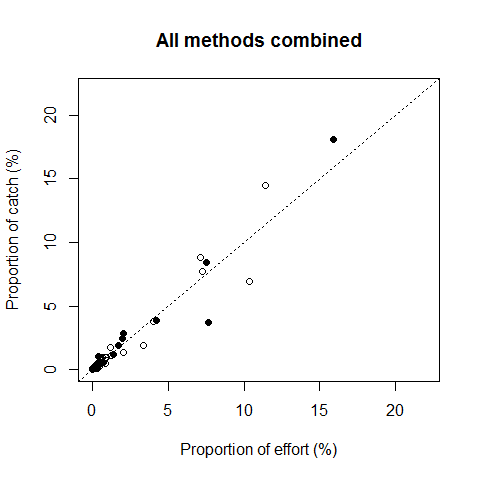

Supplement: Supplementary file 2 — High Resolution Image (TIF 673 kb) [file 10745_2016_9805_MOESM_ESM.tif]
